# EANet: Depth Estimation Based on EPI of Light Field

**DOI:** 10.1155/2021/8293151

**Published:** 2021-12-28

**Authors:** Yunzhang Du, Qian Zhang, Dingkang Hua, Jiaqi Hou, Bin Wang, Sulei Zhu, Yan Zhang, Yun Fang

**Affiliations:** ^1^School of Information, Mechanical and Electrical Engineering, Shanghai Normal University, Shanghai 200234, China; ^2^School of Computer and Remote Sensing Information Technology, North China Institute of Aerospace Engineering, Langfang 065000, China; ^3^Mathematics & Science College, Shanghai Normal University, Shanghai 201418, China

## Abstract

The light field is an important way to record the spatial information of the target scene. The purpose of this paper is to obtain depth information through the processing of light field information and provide a basis for intelligent medical treatment. In this paper, we first design an attention module to extract the features of light field images and connect all the features as a feature map to generate an attention image. Then, the attention map is integrated with the convolution layer in the neural network in the form of weights to enhance the weight of the subaperture viewpoint, which is more meaningful for depth estimation. Finally, the obtained initial depth results were optimized. The experimental results show that the MSE, PSNR, and SSIM of the depth map obtained by this method are increased by about 13%, 10 dB, and 4%, respectively, in some scenarios with good performance.

## 1. Introduction

By capturing the stereo information in a specific scene, we can get accurate spatial information. This information is of great significance for evaluating the effect of treatment and rehabilitation. The light field depth information reflects the precise spatial information of the corresponding target. Depth image acquisition is the key technology to determine whether the light field image will be widely used, and it also plays a major role in 3D reconstruction [1], target recognition [2], and other fields [3].

At present, the light field depth estimation algorithm is mainly divided into nonlearning-based methods and learning-based methods. Nonlearning methods mainly include focusing and defocusing fusion methods and stereo matching-based methods. Focusing and defocusing fusion methods can get the corresponding depth by measuring the ambiguity of pixels at different focal stacks. Lin et al. [4] used that nonoccluding pixels exhibit symmetry along the focal depth dimension centered at the in-focus slice. They gave the difference between the synthesized focal stack the hypothesized depth map and that from the LF. Tao et al. [5] proposed an optimization framework that estimates both general lightings in natural scenes and shading to improve depth regularization. Depth maps obtained by the above methods can retain more details but will introduce defocusing errors and reduce the accuracy of depth maps.

Light field image is obtained by multiple cameras shooting the same scene from different perspectives. Therefore, the depth estimation problem can be transformed into a multiview stereo matching problem. Jeon et al. [6] proposed the subpixel level multiview stereo matching algorithm based on cost volume. Wang et al. [7] constructed an occlusion model and proposed the consistency principle of angle edge and spatial image edge and combined it with Canny edge detection operator to divide occluded and nonoccluded regions; the MRF model was used to obtain the depth map. Zhu et al. [8] deduced the consistency of occluder in spatial and angle spaces and selected the nonoccluded view for each candidate occlusion point. They established the antiocclusion energy function to regularize the depth map. Lee and Park [9] proposed a depth model that estimates disparity is represented by the complex number.

In recent years, deep learning has made great achievements in the field of depth estimation. Feng et al. [10] proposed a dual-stream network to learn to estimate the disparity of multiple correlated neighborhood pixels from their epipolar plane image (EPI). The network is used to learn the weight of EPI, and the output of the two streams is combined for disparity estimation. Jeon et al. [11] designed a pipeline to determine image consistency. A learning-based framework is designed to retrieve the best cost measure and the best depth tag. Huang [12] devised a stereo matching algorithm to employ this framework on dense, sparse, and even denoised light fields. Li and Jin [13] proposed a depth foreground estimation algorithm based on the neighborhood distribution in sheared epipolar plane images (EPIs) to solve the problem of foreground occlusion. Ana et al. [14] proposed a learning-based framework making use of dilated convolution, densely connected convolutional modules, compact decoder, and skip connections.

In this paper, we mainly do the following work: (1) constructing the attention module. The SSP model is used to extract the features of the input pictures, and the features are input into the full connection layer and the convolution layer to calculate the weight of the pictures from different angles. (2) Assigning the weight to the corresponding picture to make it play different importance in light field depth estimation and (3) building a multistream network for depth estimation, learning all the input EPI clues of light field pictures, and getting the final depth estimation results. The experimental results show that the MSE, PSNR, and SSIM of the depth map obtained by this method are increased by about 13%, 10 dB, and 4%, respectively, in some scenarios with good performance.

The rest of this paper is organized as follows: in Section 2, we introduce the principle of light field and EPI and review the related work.  Section 3 proposes EANet and the attention module.  Section 4 shows the experimental results on the light field datasets. Finally, the paper is concluded in Section 5.

## 2. Related Work

### 2.1. Disparity Estimation in 4D Light Field

**Figure 1:**
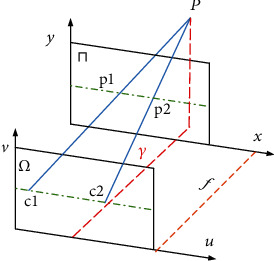
4D light field schematic.

**Figure 2:**
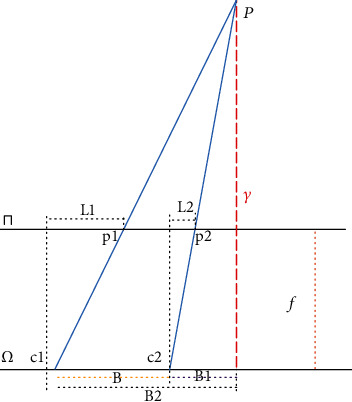
Relationship between disparity and depth.

The light field can be represented by a four-dimensional function *L* (*u*, *v*, *x*, *y*). As shown in Figures 1 and 2, the light in the light field is parameterized by intersecting plane (*u*, *v*) and (*x*, *y*).

In Figure 1, the point *P* is the space point, the plane *Π* is the camera plane, and the plane *Ω* is the imaging plane. Obviously, the required 4D light field depth *γ* and the relative position of point *P* in the two planes have a clear geometric relationship, as shown in formula (1).

(1) $$\begin{array}{c} \gamma =\left|\frac{f\left(B_{1}-B_{2}\right)}{L_{1}-L_{2}}\right|, \end{array}$$

where *γ* is the depth of point *P*, *B*_1_ and *B*_2_ are the distance between the image position of point *P* in the *Π* plane and the central viewing angle, respectively, and *L*_1_ and *L*_2_ are the distance between the point where the light of point *P* passes through the *Ω* plane and the central point of the respective subaperture viewing angle. Accurate depth information *γ* can be obtained by calculating the disparity ∣*L*_1_ − *L*_2_∣ of point *P* on the *Π* plane.

### 2.2. EPI

Epipolar plane image (EPI) contains spatial and angular information of two-dimensional slices of the light field images. EPI lines with different slopes are formed from projections of the same point at different angles. By calculating the slope of such a line in EPI, we can obtain the parallax of the pixels in the image.

**Figure 3:**
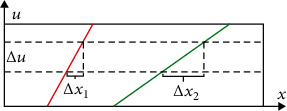
Slope of tangent line of EPI structure.

As shown in Figure 3, the Δ*u* is disparity. The relationship between ∆*u* and *γ* in Figure 2 can be expressed as formula (2).

(2) $$\begin{array}{c} \gamma =‐\frac{\text{f}}{Z}\Delta u. \end{array}$$

### 2.3. EPI-Based Depth Estimation Method

Feng et al. [10] constructed a shallower CNN and the output of the fully connected layer. Jiang et al. [15] used a fine-tuned- flow-based network to estimate the initial depth and then refined the initial result with a multiviewpoint stereo refined network. Shin et al. [16] proposed an end-to-end network to predict depth, which takes as input multiple directions viewpoints instead of EPI patches. Li and Jin [13] proposed a depth foreground estimation algorithm based on the neighborhood distribution in sheared epipolar plane images (EPIs) to solve the problem of foreground occlusion. Li et al. [17] designed a multiscale aggregated light field depth estimation network, which greatly improves the calculation speed and reduces the network complexity. Zhang et al. [18] proposed a novel method for 4D light field (LF) depth estimation exploiting the special linear structure of an epipolar plane image (EPI) and locally linear embedding. Wang et al. [19] proposed an enhanced rotation parallelogram operator based on color constraint and histogram integral (spo-ch).

However, the above studies did not consider that the repetition of structures in light field images, the redundancy of multiview information, and the importance of each subpore viewpoint in depth estimation are different.

Our paper proposes the EANet network architecture based on the study of the EPI structure of light field images and the attention mechanism in deep learning. We design an attention module in the multistream convolutional neural network. The prediction results of the new module are used to increase the weight of images, which are more valuable in depth estimation. This method is evaluated on the HCI light field datasets, and the results show that the accuracy of the light field depth estimation has been improved.

## 3. Proposed Method

Based on the above analysis, this paper proposes a depth estimation method based on the EANet (EPI-Attention-net). Firstly, we used the new module to predict the attention map. At the same time, we convoluted the light field images in the four directions separately, extracted the EPI features, and connected them. Nextly, the connected EPI feature was integrated with the attention map and learning continued. Eventually, we obtained the optimized light field depth estimation results.

### 3.1. EANet Model

**Figure 4:**
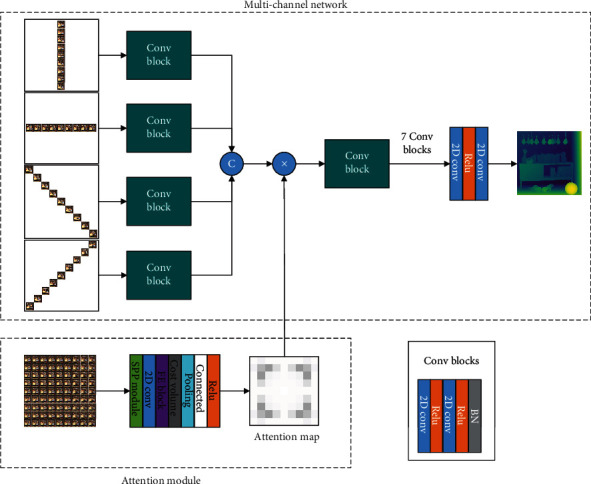
EANet network structure.

The network model proposed in this paper is shown in Figure 4. We first preprocess the input light field image and EPI information and encode the image according to the mapping relationship between EPI information and depth information. Next, a multilayer neural network is set up in the part after the multichannel network fusion. The neural network will learn the EPI information contained in the input image data during training and combine it with the prediction results obtained by the attention module.

In the multichannel network, we set up three conv-blocks, and the setting of each block is shown in the lower right corner of Figure 4. This part of the network extracts EPI structural features of light field information in different directions. After getting the EPI features in four directions, we connect them.

On the other hand, we use the attention module to process all the light field images and get the attention map. The model mainly includes the SPP layer, FE block, cost volume, and full connection layer.

After obtaining the multidirectional EPI features and attention map, we connect and merge them to learn in the subsequent network. We set up eight conv-blocks in the subsequent network and add an optimized block (composed of two 2D conv and a Relu layer) at the end.

The neural networks in this model all use sequential models to connect the convolutional layers and activation function layers in the network. This model is characterized by single input and output, with only adjacent relations between layers, and no cross-layer connections. The convolutional layers shown in Figure 4 are all 2D convolution operations. We set the size of the convolution kernel to 2 × 2 and the step size to 1. The activation function adopts the linear rectification function (Relu), and the Relu function is shown as formula (3).

(3) $$\begin{array}{c} \text{f}\left(x\right)=\max\left(0,W^{T}X+b\right), \end{array}$$

where linear rectification is used as the activation function of the neuron, which introduces a nonlinear output to the output (*w*^*T*^*x* + *b*) of the neuron in the upper layer, and *f* (*x*) is the output to the next convolutional layer. The linear rectification function (Relu) avoids the problems of gradient explosion and gradient disappearance to a certain extent.

Since the deep neural network is a multilayer overlay, it will reduce the learning speed. Moreover, the changes in the input of the lower layer tend to become larger or smaller, causing the upper layer to fall into the saturation zone, making the learning stop prematurely. Therefore, we choose batch normalization (BN) after the last activation of the function layer. As shown in formula (4),

(4) $$\begin{array}{c} \text{h}=\text{f}\left(\text{g}\cdot \frac{X-\mu }{\sigma }+\text{b}\right), \end{array}$$

where *μ* is the translation parameter and *σ* is the scaling parameter. These two parameters are used to translate and scale the data so that the data conforms to a standard distribution with a mean of 0 and a variance of 1. *b* is the retranslation parameter, and *g* is the rescaling parameter to ensure that the expressive ability of the model does not decrease due to standardization.

### 3.2. Attention Module

In recent years, the attention mechanism has become more and more widely used in the field of artificial intelligence. The 4D light field image contains a large number of subaperture viewpoints with different viewing angles. These subaperture viewpoints contain abundant parallax information, but they also have a lot of redundant information.

Tsai et al. [20] designed a light field depth estimation network by constructing a deep-level complex neural network, combining residual network with an attention mechanism. Their work combines the current mainstream methods of in-depth learning.

However, there are still some deficiencies, such as not using the EPI structure in the light field to handle all perspectives, which makes the network structure too complex, leads to computational complexity and runtime too long to be well applied in even feedback scenarios. Inspired by it, we introduce an attention module to mark the more important views for depth estimation from the light field image.

**Figure 5:**
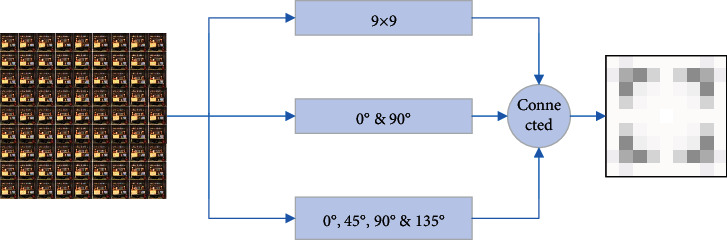
Attention module indication.

As shown in Figure 5, the attention module generates attention images, which reflect the importance of the light field images of each scene to the depth estimation results. The attention module has three modes. In the first mode, we perform attention evaluation on each image; in the second mode, only the 0° and 90° direction images are used for mirroring calculation; in the last mode, add 45° and 135° direction. Three methods are used together to get the attention map. We integrate the attention map with the convolutional layer in the neural network in the form of weight and then strengthen the weight of the subaperture viewpoints which are more meaningful for depth estimation.

**Figure 6:**
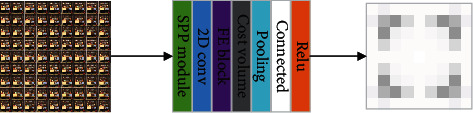
Attention module structural design.

As shown in Figure 6, specifically, first, we convoluted the light field images, preprocessing them. Then, feature extraction was performed in the SSP module, and textured areas and nonlambdoid surfaces were excluded. FE block extracts features based on the connections of neighboring regions and connects all the feature maps to obtain an output feature map. Next, in cost volume, we adjust the relative position of feature views, calculating the five-dimensional (batch size × disparity × height × width × feature dimension) cost after these feature maps are connected. Finally, the input cost volume is pooled to generate an attention map, followed by a connectivity layer and an activation layer. Take the HCI dataset as an example, with 9 × 9 subaperture viewpoints in each scene, so we end up with 9 × 9 attention maps.

Compared with other networks, our network learns light field depth information from different perspectives, and EPI information from different perspectives is complementary to each other. At the same time, the attention module is used to preprocess the data to improve the accuracy of depth estimation.

## 4. Experimental Results

In the network, we randomly sampled light field images and patch-wise training, with the size of 23 × 23. The batch size is 16, and the learning rate is 1*e*-6, using the Rmsprop optimizer.

**Figure 7:**
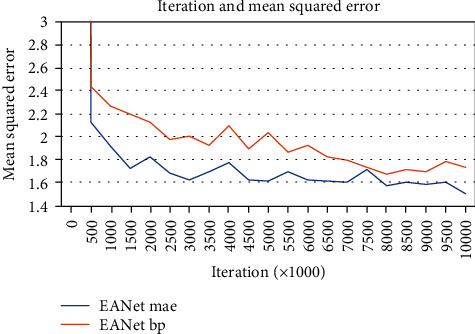
EANet iteration.

As shown in Figure 7 and Table 1, Figure 7 shows the change curve of EANet with the number of iterations, and Table 1 shows the parameters of the number of iterations at some nodes (data per thousand times in the table). When the number of iterations reached around 10000, the BP error and network MAE (mean absolute error) base were stable.

The experiment in this paper is carried out on the HCI light field dataset, which is convenient for performance comparison with other methods tested on this dataset. As part of the nonlambdoid surface is contained in the light field dataset, there are also scenarios where there are untextured regions with very small disparity. We excluded these interfering cases.

### 4.1. Subjective Analysis

**Figure 8:**
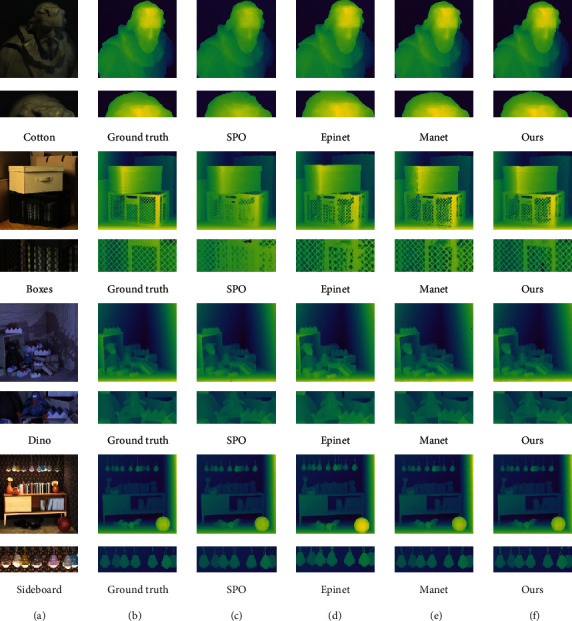
Experimental results of HCI. In each group of pictures, the first line is the overall view, and the second line is the partial enlarged picture.

The results of our method are shown in Figure 8. Figure 8(a) is the LF center view of the light field image, Figure 8(b) is the ground truth of the scene in the light field data, Figure 8(c) is the result of SPO (Zhang et al. [21]), Figure 8(d) is the result of Epinet (Shin et al. [16]), Figure 8(e) is the result of Manet, and Figure 8(f) is the result of our method.

In the result images, the depth of the color represents the distance in the light field. The lighter the color, the closer the depth. Through comparison, we can find that our method is good in detail. For example, the grid part of the boxes scene fully maintains the structure of the angle information. In button and sideboard, edge information is also well reflected. But there is little difference with other methods in the nontextured region. The specific quantization parameters are compared in the next section.

### 4.2. Quantitative Evaluation

**Figure 9:**
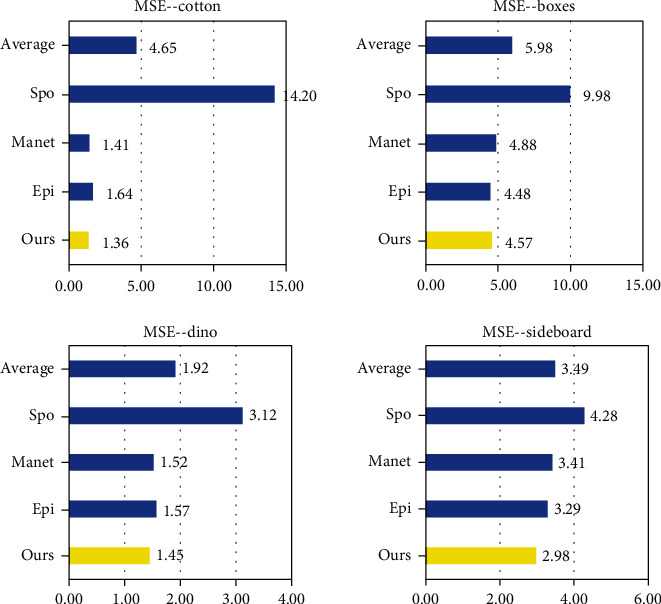
MSE (%) of results on HCI dataset.

For each method in Figure 8, we calculated their MSE, PSNR, and SSIM to evaluate the performance of each method. The MSE (mean squared error) is calculated by formula (5).

(5) $$\begin{array}{c} \text{MSE}=\frac{1}{N}\overset{1}{\underset{N}{\sum }}{\left(\text{GT}\left(i\right)-\text{Dep}\left(i\right)\right)}^{2}, \end{array}$$

where *N* is the total number of pixels in the depth map and Dep and GT represent the final depth map and ground truth of light field, respectively. The *i* represents each pixel in the image.  Table 2 and Figure 9 show the MSE of light field scenes in Figure 8 of SPO, Epinet, Manet, and ours. Our method has been greatly improved in cotton by about 13%.

**Figure 10:**
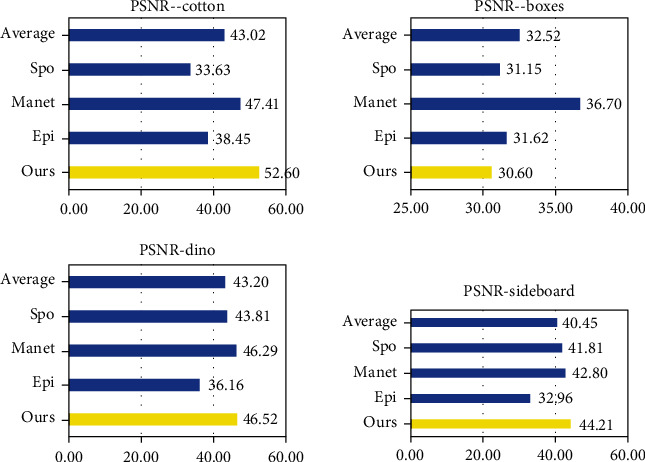
PSNR (dB) of results on HCI dataset.

PSNR is the most widely used objective image evaluation index based on the error between corresponding pixels. The PSNR (peak signal-to-noise ratio) is calculated by formula (6).

(6) $$\begin{array}{c} \text{PSNR}=10\cdot \log_{10}\left(\frac{{\max_{I}}^{2}}{\text{MSE}}\right), \end{array}$$

where max_*I*_ is the maximum value of pixels in the image.  Table 3 and Figure 10 show the PSNR of light field scenes.

**Figure 11:**
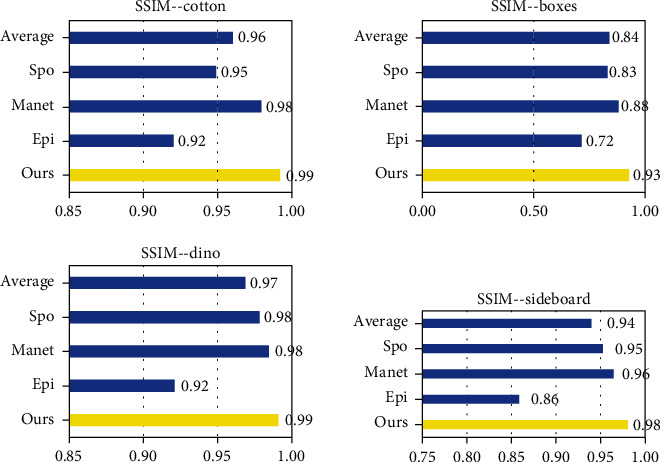
SSIM of results on HCI dataset.

The SSIM (structural similarity) is an index to measure the similarity of two images. Comparing GT with a depth map can reflect the accuracy of depth estimation. The SSIM is calculated by formula (7).

(7) $$\begin{array}{c} \text{SSIM}\left(x,y\right)=\frac{\left(2\mu_{x}\mu_{y}+c_{1}\right)\left(2\sigma_{xy}+c_{2}\right)}{\left(\mu_{x}^{2}+\mu_{y}^{2}+c_{1}\right)\left(\sigma_{x}^{2}+\sigma_{y}^{2}+c_{2}\right)}, \end{array}$$

where *x* and *y* represent depth map and ground truth, respectively, *μ* is the mean of the image, *σ*_*x*_^2^ and *σ*_*x*_^2^ are the variance of the image, *σ*_*x*,*y*_  is the covariance of *x* and *y*, and c is a constant term.  Table 4 and Figure 11 show the SSIM of light field scenes. It can be seen from Figure 10 that our method has improved in all scenarios, about 4%.

By observing the results in Tables 2–4, we can draw the following conclusion: Table 1 shows that in most cases, we achieved better MSE performance. In sideboard, compared with Epinet and Manet, the MSE of this method is reduced by 0.31 and 0.38 on average. In Table 3, our method has been greatly improved in cotton and sideboard, about 13%. It can be seen from Figure 9 that our method has improved in all scenarios, about 9.26%. In Table 4, our SSIM is also significantly higher than average.

### 4.3. Ablation Experiment

To show the improvement of the EANet by our method, we designed a corresponding ablation experiment.

**Figure 12:**
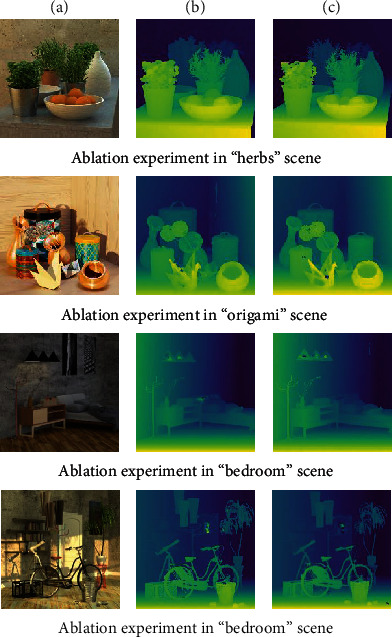
Ablation experiment.

We conducted experiments on “herbs,” “origami,” “bedroom,” and “bicycle.” Figure 12 is a comparison of the result images, and Table 5 is the comparison of MSE, PSNR, and SSIM in four scenarios. Figure 12(a) is the center view of each scene, and Figures 12(b) and 12(c) are the results of whether to add the attention model.

In Table 5, we use “√” and “×” to indicate whether the attention model is useful or not. Our method reduces the MSE by about 10%, increases the PSNR by 0.3 dB, and SSIM by 0.03%. Compared with Figure 11 and Table 4, our method can better extract and save the edge details and angle information of the image in the depth estimation process, improve the accuracy of the depth map, and reduce the error.

## 5. Conclusion

In this paper, we propose a depth estimation network based on the attention module and light field EPI cues. First, the importance of the light field images is predicted by focusing on the module. Then, the prediction results are combined with the depth estimation network. Finally, accurate depth information is obtained. This network has both accuracy and computational efficiency. It calculates the importance of images while fully considering the angle of light field and EPI information and further explores the value of pictures from different perspectives. Our approach achieves competitive results in visual quality, PSNR, and SSIM.

The work in this paper has laid the research foundation for the subsequent development of light field reconstruction, but there are still shortcomings in many aspects, such as increasing the number of real datasets, the reflection area, and no texture. We leave this to future work.

## Figures and Tables

**Table 1:** EANet iteration data.

Iteration (1000)	1000	2000	3000	4000	5000	6000	7000	8000	9000	10000
EANet MAE	1.92	1.83	1.63	1.78	1.62	1.63	1.61	1.58	1.59	1.51
EANet BP	2.27	2.13	2.01	2.1	2.04	1.93	1.8	1.68	1.7	1.74

**Table 2:** MSE (%) of results on HCI dataset.

	Cotton	Boxes	Dino	Sideboard
Ours	1.36	4.57	1.45	2.98
Epinet [16]	1.64	4.48	1.57	3.29
Manet [17]	1.41	4.88	1.52	3.41
SPO [21]	14.2	9.98	3.12	4.28
Average	4.65	5.98	1.92	3.49

**Table 3:** PSNR (dB) of results on HCI dataset.

	Cotton	Boxes	Dino	Sideboard
Ours	52.60	30.60	46.52	44.21
Epinet [16]	38.45	31.62	36.16	32.96
Manet [17]	47.41	36.70	46.29	42.80
SPO [21]	33.63	31.15	43.81	41.81
Average	43.02	32.52	43.20	40.45

**Table 4:** SSIM of results on HCI dataset.

	Cotton	Boxes	Dino	Sideboard
Ours	0.99	0.93	0.99	0.98
Epinet [16]	0.92	0.72	0.92	0.86
Manet [17]	0.98	0.88	0.98	0.96
SPO [21]	0.95	0.83	0.98	0.95
Average	0.96	0.84	0.97	0.94

**Table 5:** Ablation experiment.

Scene	Module	MSE (%)	PSNR (dB)	SSIM
Herbs	×	1.46	26.5	0.42
	√	1.37	26.77	0.43

Origami	×	1.69	25.83	0.51
	√	1.6	26.09	0.56

Bedroom	×	0.87	28.7	0.49
	√	0.86	28.77	0.5

Bicycle	×	1.39	26.69	0.25
	√	1.34	26.85	0.29

## Data Availability

The raw/processed data required to reproduce these findings cannot be shared at this time as the data are also part of an ongoing study. The datasets generated and/or analyzed during the current study are available from the corresponding author on reasonable request.

## References

[1] Waller L., Tian L. (2015). 3d Intensity And Phase Imaging From Light Field Measurements In An Led Array Microscope. *Optica*.

[2] Xu Y., Maeno K., Nagahara H., Shimada A., Taniguchi R. I. (2015). Light Field Distortion Feature For Transparent Object Classification. *Computer Vision and Image Understanding*.

[3] Xie B., Yang J., Shen J., Lv Z. Image Defogging Method Combining Light Field Depth Estimation And Dark Channel.

[4] Lin H., Chen C., Bing Kang S., Yu J. Depth Recovery From Light Field Using Focal Stack Symmetry.

[5] Tao M. W., Srinivasan P. P., Malik J., Rusinkiewicz S., Ramamoorthi R. Depth From Shading, Defocus, And Correspondence Using Light-Field Angular Coherence.

[6] Jeon H.-G., Park J., Choe G. Accurate Depth Map Estimation From A Lenslet Light Field Camera.

[7] Wang T.-C., Efros A. A., Ramamoorthi R. (2016). Depth Estimation With Occlusion Modeling Using Light-Field Cameras. *IEEE Transactions on Pattern Analysis and Machine Intelligence*.

[8] Zhu H., Wang Q., Yu J. (2017). Occlusion-Model Guided Antiocclusion Depth Estimation In Light Field. *IEEE Journal of Selected Topics in Signal Processing*.

[9] Lee J. Y., Park R. H. (2021). Complex-Valued Disparity: Unified Depth Model Of Depth From Stereo, Depth From Focus, And Depth From Defocus Based On The Light Field Gradient. *IEEE Transactions On Pattern Analysis And Machine Intelligence*.

[10] Feng M., Wang Y., Liu J., Zhang L., Zaki H. F. M., Mian A. (2018). Benchmark Data Set And Method For Depth Estimation From Light Field Images. *IEEE Transactions on Image Processing*.

[11] Jeon H.-G., Park J., Choe G. (2019). Depth From A Light Field Image With Learning-Based Matching Costs. *IEEE Transactions on Pattern Analysis and Machine Intelligence*.

[12] Huang C.-T. (2019). Empirical Bayesian Light-Field Stereo Matching By Robust Pseudo Random Field Modeling. *IEEE Transactions on Pattern Analysis and Machine Intelligence*.

[13] Li J., Jin X. Epi-Neighborhood Distribution Based Light Field Depth Estimation.

[14] Ana N., Geravand M., Braendler D., Bull D. R. Fast Depth Estimation For View Synthesis.

[15] Jiang X., Shi J., Guillemot C. A Learning Based Depth Estimation Framework For 4d Densely And Sparsely Sampled Light Fields.

[16] Shin C., Jeon H. G., Yoon Y. Epinet: A Fully-Convolutional Neural Network For Light Field Depth Estimation Using Epipolar Geometry.

[17] Yan Li L., Zhang Q. W., Lafruit G. Manet: Multi-Scale Aggregated Network For Light Field Depthe Stimation.

[18] Zhang Y., Lv H., Liu Y., Wang H., Dai Q. (2017). Light-Field Depth Estimation Via Epipolar Plane Image Analysis And Locally Linear Embedding. *IEEE Transactions on Circuits and Systems for Video Technology*.

[19] Wang W., Lin Y., Zhang S. (2021). Enhanced Spinning Parallelogram Operator Combining Color Constraint And Histogram Integration For Robust Light Field Depth Estimation. *IEEE Signal Processing Letters*.

[20] Tsai Y.-J., Liu Y.-L., Ouhyoung M., Chuang Y.-Y. (2020). Attention-Based View Selection Networks For Light-Field Disparity Estimation. *Proceedings of the AAAI Conference on Artificial Intelligence*.

[21] Zhang S., Sheng H., Li C., Zhang J., Xiong Z. (2016). Robust Depth Estimation For Light Field Via Spinning Parallelogram Operator. *Computer Vision and Image Understanding*.

